# Perinatal risk factors for pediatric onset type 1 diabetes, autoimmune thyroiditis, juvenile idiopathic arthritis, and inflammatory bowel diseases

**DOI:** 10.1007/s00431-021-03987-3

**Published:** 2021-02-23

**Authors:** Laura Räisänen, Heli Viljakainen, Catharina Sarkkola, Kaija-Leena Kolho

**Affiliations:** 1 Faculty of Medicine and Health Technology (MET), Tampere University, Tampere, Finland; 2 Folkhälsan Research Center, Helsinki, Finland; 3 Faculty of Medicine, University of Helsinki, Helsinki, Finland; 4 Faculty of Medicine and Children´s Hospital, Helsinki University Hospital, University of Helsinki, Helsinki, Finland

**Keywords:** Autoimmune diseases, Environmental risk factors, Maternal background, Perinatal conditions

## Abstract

Type 1 diabetes mellitus (DM), autoimmune thyroiditis (AIT), juvenile idiopathic arthritis (JIA), and inflammatory bowel diseases (IBD) are common pediatric autoimmune diseases with unknown risk factors. Using nationwide registers, we searched for their perinatal risk factors. Our study followed up 11,407 children (born 2000–2005) for a median of 16.6 years (from birth to 2018). Of them, 2.15% received primary diagnosis and 0.08% also secondary: 0.89% had DM, 0.60% had AIT, 0.48% had JIA, and 0.25% had IBD. The incidences per 100,000 children/year were 106.1 for DM, 46.0 for AIT, 55.0 for JIA, and 23.7 for IBD. There were more preterm births (< 37 weeks) among children with studied autoimmune diseases compared with the rest of the cohort (8.6% vs. 5.3%, *p* = 0.035). Among those born preterm, children with studied autoimmune diseases received more postnatal antibiotics compared with other preterm children in the cohort (47.6% vs. 27.7%, *p* = 0.046). Children with IBD were born to older mothers compared with those without studied diagnoses (33.0 vs 30.2, *p* = 0.004).

*Conclusion*: Preterm birth was a shared risk factor for autoimmune diseases in our study, especially when combined with postnatal antibiotic treatments. High maternal age was associated with IBD.

**Table:** 

**What is Known:** • *Type 1 diabetes (DM), autoimmune thyroiditis (AIT), juvenile idiopathic arthritis (JIA), and inflammatory bowel diseases (IBD) are common pediatric autoimmune diseases* • *It is unclear whether these diseases have shared risk factors, since there are no previous simultaneous epidemiological nor follow-up studies on them in one cohort *	
**What is New:** • *Preterm births were more common in children with DM, AIT, JIA, or IBD compared with other children in the cohort, and preterm children who developed these diseases recieved more postnatal antibiotics compared with other preterm children* • *High maternal age was associated with IBD*

**Supplementary Information:**

The online version contains supplementary material available at 10.1007/s00431-021-03987-3.

## Introduction

Autoimmune diseases are disorders, where the immune system attacks normal tissues. In genetically susceptible individuals, this could be triggered by environmental factors [1]. Genome-Wide Association Studies have uncovered multiple predisposing genes that overlap between several autoimmune diseases [2–4]. Yet, mutual environmental triggers have not been identified—even when continuously increasing incidence of these diseases (including those with pediatric onset) may suggest their presence [5].

This study focuses on four common pediatric autoimmune diseases: type 1 diabetes (DM), autoimmune thyroiditis (AIT), juvenile idiopathic arthritis (JIA), and inflammatory bowel diseases (IBD). DM is a disorder of insulin secretion due to destruction of β cells in the pancreatic islets of Langerhans by autoreactive T cells, causing hyperglycemia [6–8]. AIT is a disorder of thyroid hormone secretion due to T cell-mediated attack on thyroid gland, causing hypothyroidism [9]. JIA is a heterogeneous disease. Dysfunction of adaptive immune system in oligo-/polyarticular JIA results in accumulation of activated T cells in synovial membrane [10]. While oligo-/polyarticular JIA stays in the joints, systemic JIA (presenting dysfunction of the innate immune system) could affect extrasynovial tissues as well, eliciting symptoms like spiking fever, rash, serositis, lymphadenopathy, and hepatosplenomegaly [11]. IBD are chronic autoimmune inflammations of gastrointestinal tract, involving T cell infiltration in the gut mucosa [12]. There are two main IBD subtypes: Crohn disease (CD) and ulcerative colitis (UC) [13, 14]. CD causes transmural inflammation and may involve any part of the gastrointestinal tract, while UC causes mucosal inflammation and is limited to the colon. When variations in the location, nature, and severity of inflammation in the colon are present, the term IBD unclassified (IBDU) is used [15].

Since DM, AIT, JIA, and IBD have similar characteristics, such as (1) overlapping genetic associations, (2) chronic and usually intermittent inflammation, (3) involvement of T cell organ infiltrations, and (4) increasing incidence without specific triggering factors, we assumed that they may have mutual environmental risk factors. This study aims to search for these risk factors right from the beginning—among maternal and perinatal backgrounds.

## Methods

### Data sources and study population

This register-based cohort study was conducted among participants of Finnish Health in Teens (Fin-HIT) study. The nationwide Fin-HIT cohort was assembled mostly by school recruitments in vast, densely populated areas throughout Finland without specific exclusion criteria. In total, 11,407 children (52.2% girls) born between 2000 and 2005 participated. Their families eligibly represented general population, even when the parent’s education level was relatively high. More details on recruitment and characteristics of the cohort were described elsewhere [16]. The frequency of DM, AIT, JIA, and IBD and their possible maternal and perinatal risk factors were studied by linking the cohort to three national health registers: Medical Birth Register (MBR), Special Reimbursement Register (SRR), and Drug Purchase Register (DPR). The excellent coverage of these registers has been described previously [17]. MBR held by the Finnish Institute for Health and Welfare (THL) provides information on maternal backgrounds (employment status before delivery, smoking habit, age, parity, and delivery method) and perinatal factors (birthweight, height, gestational length, postnatal antibiotic treatment, and inpatient length of stay) [18]. MBR was successfully linked to 11,380 children. Of them, 10,944 had available maternal and perinatal information.

### Identification of patients with chronic immune-mediated diseases in the cohort

Each Finnish resident has a personal identifier number. Every register linkage is based on this, including SRR and DPR maintained by the Finnish Social Insurance Institution [17, 19]. SRR contains records on patients with chronic diseases necessitating medications, who are entitled to drug refunds (i.e., special reimbursements) regardless of their socioeconomic status. These records include diagnoses (verified by physicians according to standardized criteria) and entry dates. DPR lists all purchases of prescribed medications, including dispensation dates and pharmaceutical information [17].

DM, JIA, and IBD diagnoses in the cohort (from birth until 31 December 2018) were obtained using ICD-10 codes (International Classification of Diseases (ICD), 10th revision) from SRR (E10.9 for DM; M08 for JIA; K50 (CD) and K51 (UC/IBDU) for IBD). AIT diagnoses were obtained from DPR using ATC (Anatomical Therapeutic Chemical) code H03AA01 for thyroxin—the only drug used for AIT, which is available on prescription only. DPR was chosen for identifying AIT because thyroxin is inexpensive and not everyone applies for the special reimbursement/is registered in SRR.

### Statistical methods

The data were analyzed through two perspectives. (1) Patients: when studied autoimmune diseases were observed concurrently, secondary diagnoses were ignored and the index group comprised all individuals with studied autoimmune diseases (*N* = 245). (2) Diagnoses: when each diagnosis (DM, AIT, JIA, or IBD) were observed individually, secondary diagnoses were considered, comprising 254 cases. When estimating the peak incidence of the studied autoimmune diseases, the study population was divided into three groups based on children’s physical developmental stages and corresponding usual daytime environment: infancy to preschool age (0–5.9 years in daycare), pre-pubertal age (6–11.9 years in primary school), and adolescence (12–18 years in secondary school).

Categorical variables were tested using Chi-square (*χ*^2^) or Fischer’s exact test. Continuous variables were tested using Independent sample *t* test or Kruskal-Wallis test. The data are displayed with number and proportion (%), mean and standard deviation (SD), or median and interquartile range (IQR). When the size and proportion of the cohort were regarded, 95% confidence interval (CI) was presented with Wilson score interval. The software used was IBM SPSS Statistics 26.0 and a 5% statistical significance level was adopted.

## Results

The cohorts of 11,407 children born in 2000–2005 were followed-up from birth until 31 December 2018. Their end-point median age was 16.6 years (IQR 14.6–18.6). Of them, 245 children (2.1%) received at least one diagnosis: 102 (0.89%) were diagnosed with DM, 68 (0.60%) with AIT, 55 (0.48%) with JIA, and 29 (0.25%) with IBD (UC 19 (0.17%) and CD 10 (0.09%)) (Table 1). Only 9 children out of 245 (0.08% of the whole cohort) were presented with multiple diagnoses—7 had DM and AIT, 1 had AIT and JIA, and 1 had DM and IBD—comprising 254 diagnoses.

**Table 1:** Prevalence, incidence per 100,000 children/year, and median age of diagnosis of pediatric onset DM, AIT, JIA, and IBD^a^. The 11,407 children in the cohort were born in 2000–2005 and follow-up was continued until a median age of 16.6 years. Of them, 245 received primary diagnoses and 9 received secondary diagnoses as well

Diagnosis	Prevalence (*N* = 254) (2.23% of total cohort)	Incidence/100,000 children/year (95% CI^b^)	Median age of diagnosis (IQR^c^)
DM	102 (0.89%)	106.1 (87.5–128.6)	8.6 (4.8–11.9)
AIT	68 (0.60%)	46.0 (36.3–58.2)	13.9 (11.1–15.8)
JIA	55 (0.48%)	55.0 (42.3–71.6)	9.0 (4.4–12.9)
IBD	29 (0.25%)	23.7 (16.5–34.0)	11.6 (8.3–13.6)

^a^Abbreviations: *DM* type 1 diabetes mellitus, *AIT* autoimmune thyroiditis, *JIA* juvenile idiopathic arthritis, *IBD* inflammatory bowel diseases

^b^Confidence interval using Wilson score interval

^c^Interquartile range

**Fig. 1:**
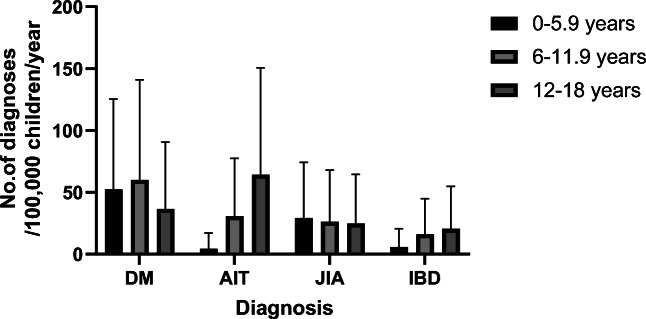
Incidence of pediatric onset DM, AIT, JIA, and IBD^a^ per 100,000 children per year with upper 95% CI. The children were born in 2000–2005 and follow-up was continued until a median age of 16.6 years. Legend: ■ = 0–5.9 years,  = 6–11.9 years,  = 12–18 years. ^a^ Abbreviations: DM = type 1 diabetes mellitus, AIT = autoimmune thyroiditis, JIA = juvenile idiopathic Arthritis, IBD = Inflammatory bowel diseases

**Fig. 2:**
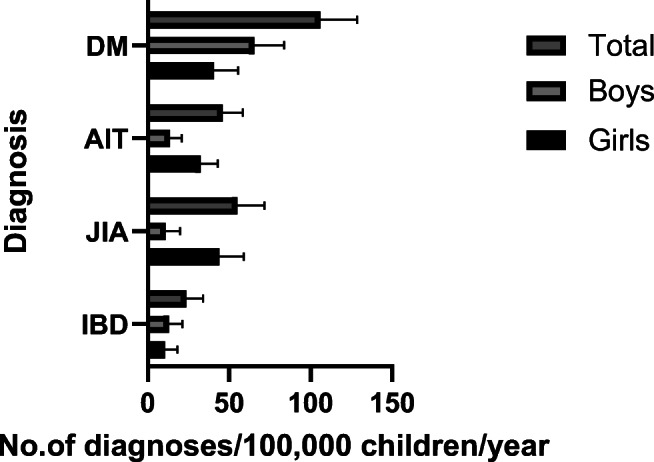
Sex distribution of DM, AIT, JIA, and IBD^a^ per 100,000 children per year with upper 95% CI. The children were born in 2000–2005 and follow-up was continued until a median age of 16.6 years. Legend:  = total,  = boys, ■ = girls . ^a^ Abbreviations: DM = type 1 diabetes mellitus, AIT = autoimmune thyroiditis, JIA = juvenile idiopathic arthritis, IBD = inflammatory bowel diseases

Of the four diagnoses, DM had the highest incidence (106.1/100,000 children/year) and IBD had the lowest (23.7; UC 16.3 and CD 7.5) (Table 1). AIT had the oldest median age of diagnosis (13.9 years, IQR 11.1–15.8), while DM had the youngest (8.6 years, IQR 4.8–11.9). DM and JIA emerged equally in different age groups (Fig. 1, Supplement Table 1). By contrast, AIT was significantly highest during adolescence, reaching 64.5 (95% CI 48.1–86.5), lower in pre-pubertal age (30.8; 95% CI 20.1–48.1), and lowest in preschool age (4.4; 95% CI 1.5–12.9). The peak incidence of IBD was also seen during adolescence with 20.5 (95% CI 12.2–34.4), but the difference was not prominent compared with other age groups: 16.1 (95% CI 9.0–28.9) for pre-pubertal age and 5.9 (95% CI 2.3–15.1) for preschool age. DM was more common in boys, in contrast to AIT and JIA (Fig. 2, Supplement Table 2). IBD were equally distributed between both sexes.

Children with studied autoimmune diseases experienced more preterm births (born < 37 weeks) than the rest of the cohort (8.6% vs. 5.3%, *p* = 0.035) (Table 2). Of all preterm children in the cohort (*N* = 614), 42.7% were born by cesarean section (15.4% in full term, *p* < 0.001) and 28.3% received postnatal antibiotics (3.2% in full term, *p* < 0.001). Furthermore, preterm children born by cesarean section (*N* = 262) received more postnatal antibiotics compared with preterm children born by vaginal delivery (55.2% vs. 44.8%, *p* < 0.001), implying for a potential link between preterm birth, cesarean section, and early antibiotic use in general.

**Table 2:** Frequency of DM, AIT, JIA, and IBD^a^ in relation with maternal backgrounds, delivery method, and perinatal factors. Of 11,407 children in the study population, 245 received primary diagnoses and 9 received secondary diagnoses as well. Medical Birth Register was available for 11,380 children

Diagnosis	DM (*N* = 102)	AIT (*N* = 68)	JIA (*N* = 55)	IBD (*N* = 29)	Children with primary diagnoses (*N* = 245)	Children without diagnoses (*N* = 11,135)	*p* value
Maternal employment status (%)							0.655^b^
- Upper-level employees	35 (34.3)	13 (19.1)	14 (25.5)	10 (34.5)	71 (29.0)	3096 (27.8)	
- Lower-level employees	38 (37.2)	33 (48.5)	20 (36.4)	10 (34.5)	97 (39.6)	4199 (37.7)	
- Manual and self-employed workers	9 (8.8)	7 (10.3)	10 (18.2)	4(13.8)	28 (11.4)	1375 (12.3)	
- Students	6 (5.9)	7 (10.3)	6 (10.9)	3 (10.3)	21 (8.6)	1106 (9.9)	
- Housewives	7 (6.9)	4 (5.9)	4 (7.3)	2 (6.9)	17 (6.9)	628 (5.6)	
- Unemployed, pensioners	0	1 (1.5)	0	0	1 (0.4)	31 (0.3)	
- Missing data	7 (6.9)	3 (4.4)	1 (1.8)	0	10 (4.0)	701 (6.3)	
Mother’s smoking habit (%)							0.825^b^
- Not smoking	87 (85.3)	59 (86.8)	49 (89.1)	26 (89.7)	213 (86.9)	9333 (83.8)	
- Quitted during 1st trimester	3 (2.9)	1 (1.5)	2 (3.6)	0	6 (2.4)	190 (1.7)	
- Smoked after 1st trimester	7 (6.9)	7(10.2)	2 (3.6)	2 (6.9)	17 (6.9)	835 (7.5)	
- Missing data	5 (4.9)	1 (1.5)	2 (3.6)	1 (3.4)	9 (3.7)	778 (7.0)	
Maternal age, mean ± SD	31.0 ± 5.3	30.5 ± 5.3	29.8 ± 5.5	33.0 ± 5.1^c*^	30.9 ± 5.3	30.2 ± 5.2	0.480^c^
- Missing data (%)	2 (2.0)	1 (1.5)	0	0	3 (1.2)	471 (4.2)	
Children’s birth year (%)							0.316^b^
- 2000	16 (15.7)	14 (20.6)	9(16.4)	3(10.3)	41 (16.7)	1587 (14.3)	
- 2001	24 (23.5)	20 (29.4)	8(14.5)	10 (34.5)	59 (24.1)	2498 (22.4)	
- 2002	31 (30.4)	29 (42.6)	24 (43.6)	10 (34.5)	90 (36.7)	3513 (31.5)	
- 2003	9 (8.8)	2 (2.9)	8(14.5)	2 (6.9)	20(8.2)	1038(9.3)	
- 2004	10(9.8)	1 (1.5)	4 (7.3)	4(13.8)	19(7.8)	1190 (10.7)	
- 2005	10(9.8)	1 (1.5)	2 (3.6)	0	13(5.3)	851 (7.6)	
- Missing data	2 (2.0)	1 (1.5)	0	0	3 (1.2)	458 (4.1)	
Parity, median	1	1	1	1	1	1	0.540^d^
(IQR)	(0–2)	(0–2)	(0–2)	(0–3)	(0–2)	(0–2)	
(Range)	(0–3)	(0–4)	(0–11)	(0–6)	(0–11)	(0–14)	
- Missing data (%)	3 (2.9)	1 (1.5)	0	0	4 (1.6)	477 (4.3)	
Delivery method (%)							0.430^b^
- Normal vaginal delivery	77 (75.5)	53 (77.9)	45 (81.8)	19 (65.5)	186 (75.9)	8125 (73.0)	
- Forceps or vacuum extraction	3 (2.9)	5 (7.4)	3 (5.5)	3 (10.3)	14 (5.7)	710 (6.4)	
- Cesarean section	19 (18.6)	9(13.2)	7(12.7)	7 (24.1)	41 (16.7)	1805 (16.2)	
- Missing data	3 (2.9)	1 (1.5)	0	0	4 (1.6)	496 (4.5)	
Birthweight (g), mean ± SD	3471 ± 573	3393 ± 575	3394 ± 593	3553 ± 628	3440 ± 590	3515 ± 559	0.993^c^
- Missing data (%)	3 (2.9)	1 (1.5)	0	0	4 (1.6)	474 (4.3)	
Birth height (cm), mean ± SD	49.9 ± 2.8	49.8 ± 2.5	49.9 ± 2.9	50.5 ± 2.8	49.9 ± 2.7	50.2 ± 2.5	0.905^c^
- Missing data (%)	3 (2.9)	1 (1.5)	0	0	4 (1.6)	507 (4.6)	
Length of gestation (%)							0.035^b^
- ≥ 37 weeks	90 (88.2)	63 (92.6)	50 (90.9)	25 (86.2)	219 (89.4)	10,044 (90.2)	
- < 37 weeks	9 (8.8)	4 ( 5.9)	4 (7.3)	4(13.8)	21 (8.6)	593 (5.3)	
- Missing data	3 (2.9)	1 (1.5)	1 (1.8)	0	5 (2.0)	499 (4.5)	
Postnatal antibiotic treatments (%)	5 (5.4)	3 (4.5)	5 (9.1)	3 (10.3)	16 (6.6)	487 (4.6)	0.135^b^
- Missing data	2 (2.0)	1 (1.5)	0	0	3 (1.2)	471 (4.2)	
Inpatient length of stay (days), median (range)	3 (1–39)	3 (2–12)	3 (2–9)	3 (2–7)	3 (1–39)	3 (0–71)	0.978^d^
- Missing data (%)	3 (2.9)	1 (1.5)	0	0	4 (1.6)	479 (4.3)	

^a^*DM* = type 1 diabetes mellitus, *AIT* = autoimmune thyroiditis, *JIA* = juvenile idiopathic arthritis, *IBD* = inflammatory bowel diseases

^b^Chi-square test for children with primary diagnoses and without diagnoses

^c^Independent sample *t* test for children with primary diagnoses and without diagnoses

^c*^When compared with children without diagnoses using independent sample *t* test, *p* = 0.004

^d^Kruskall-Wallis *H* test for children with primary diagnoses and without diagnoses

When focusing on preterm children only (*N* = 614), most maternal and perinatal factors were similar in those who developed studied autoimmune diseases and in those who did not (Supplement Table 3a). Birth by cesarean section was also as common in those who developed studied autoimmune diseases and in other preterm children (47.6% vs. 42.5%, *p* = 0.581) (Supplement Table 3a). In contrast, preterm children who developed studied autoimmune diseases received postnatal antibiotic treatments more frequently compared with other preterm children (47.6% vs. 27.7%, *p* = 0.046). This finding was seen only in concurred analysis of the studied autoimmune diseases and was not associated with any particular autoimmune diagnosis (Supplement Table 3b). In full term children (born ≥ 37 weeks), postnatal antibiotic treatments were equally low in children who developed autoimmune diseases and in those who did not (2.1% vs. 1.8%, *p* = 0.692).

The mothers of IBD children had higher maternal age compared with the mothers of children without studied autoimmune diseases (33.0 vs 30.2 years, *p* = 0.004). Other maternal and perinatal factors were similar in children with any of the studied autoimmune diseases and in those without these diseases.

## Discussion

Our study is the first to describe the simultaneous prevalence of DM, AIT, JIA, and IBD in a pediatric cohort. The 11,407 children in our study covered 3.5% of all Finnish children born in 2000–2005 [20]. After a median follow-up of 16.6 years, 2.1% obtained above diagnoses—mostly established at the age of 12–18 years (especially AIT and IBD). These children experienced more preterm births (< 37 weeks) compared with the rest of the cohort (8.8% vs. 5.6%). Furthermore, among those born preterm, more frequent use of postnatal antibiotics was associated with above autoimmune diseases.

The frequencies of many pediatric onset autoimmune diseases have been escalating especially in the western world. Finland is no exception. While the incidence of DM in Sweden, Norway, and UK-Northern Ireland were over 30.0 in 2009–2013, in our study (2000–2018) it reached up to 106.1/100,000 children/year [21]. The prevalence of AIT in our study was 0.60%—over four times higher than in Scotland (1993–1995), with 0.135% for individuals under 22 years [22]. Furthermore, our incidence of pediatric AIT was 46.0—almost three times higher than in a Spanish study (2010–2016) with 16.7 in children under 15 years [23]. As for JIA, the incidence in our study was 55.0, higher than in previous decades (19.5 in 1995) [24]. JIA incidence in other Scandinavian countries (1997–1999) ranged between 7 and 23, and in USA (1996–2006) it was 11.9 [25, 26]. The situation with IBD is also worrisome. Continuous increase of IBD incidence in children has been reported especially in northern Europe and North America [27, 28]. In our study, the incidence of IBD was 23.7 (16.3 for UC and 7.5 for CD), which corresponded to previous Finnish study presenting increased incidence from 7.0 in 1987–1990 to 23 in 2011–2014 [29]. In other Scandinavian countries, the annual incidence of pediatric IBD in Norway (2005–2007) was 10.9 (CD 6.8 and UC 3.6) and in Sweden (2002–2007) was 12.8 (CD 9.2 and UC 2.8) [30, 31]. Unexpectedly, Finland had the highest incidence of all four studied autoimmune diseases.

To plan effective preventive strategies, it is necessary to identify shared environmental risk factors for pediatric autoimmune diseases above. While searching for these risk factors among perinatal backgrounds, we discovered that preterm birth was a shared risk factor. Throughout Finland, 6.0% were born preterm in 2000–2005 [32]. Even though preterm birth in our study was not directly associated with any particular autoimmune disease, the highest preterm frequency (13.7%) was observed in patients with IBD. This corresponded with previous study associating preterm births with the onset of IBD later in life [33]. Preterm births have been associated with the development of DM, AIT, and JIA as well [34–37]. Despite that many studies have successfully related preterm birth and individual autoimmune diseases above, none of them have studied them simultaneously. Therefore, our findings implied that preterm birth might be a risk factor for autoimmune diseases in general and not limited only to certain diseases.

The potential mechanism relating preterm birth and autoimmune diseases can be speculated. Preterm children generally received more postnatal antibiotics compared with full-term children (28.3% vs. 3.2% in our study), which we have presented as a potential risk factor. In addition, preterm birth may deprive neonates from influences of intrauterine factors such as maternal antibodies and placenta-derived elements, while prematurely exposing them to extrauterine factors such as microbiota and nutritional antigens [38]. Together, these may alter the development of immune responses and susceptibility to autoimmune diseases. Moreover, maternal autoimmune diagnosis may induce preterm birth, as seen in case of IBD [39]. However, in our study, maternal diagnoses were not available.

Preterm birth might also connect autoimmune diseases and high maternal age. Despite that maternal age seemed unrelated to autoimmune diseases in our study, further observation revealed that the mothers of children with IBD were on average 3 years older at delivery (33.0) compared with the mothers of children without studied autoimmune diseases (30.2). Similar finding has been presented previously at least for Crohn disease [40]. A Swedish medical birth register study showed that older mothers were more prone to preterm delivery [41]. In Finland, mean maternal age was 27.6 years in 2000 and 30.0 years in 2005, but the percentage of preterm birth has not been increasing (6.3% in 2000 and 5.7% in 2005) [32, 42]. In theory, high maternal age could be related to IBD via preterm birth, but on the other hand, since there were only 29 children with IBD in our study, it is possible that our finding on maternal age, though significant, was a coincidence.

The link between autoimmune diseases and cesarean section has been studied previously with ambiguous findings [43, 44]. In line with other studies on DM and IBD, we showed that cesarean section itself was not a risk factor for studied autoimmune diseases in full term nor in preterm children [44–47]. Instead, preterm children born by cesarean section received more postnatal antibiotics than preterm children born by vaginal delivery. This, the use of postnatal antibiotics in preterm children born by cesarean section rather than the cesarean section itself, was more tightly associated with the onset of autoimmune diseases—potentially explaining some of the ambiguous findings in previous studies. In fact, we demonstrated that preterm children who later developed DM, AIT, JIA, or IBD received 1.7-time more postnatal antibiotics compared with preterm children without these diseases. Even though our study did not confirm the role of postnatal antibiotic treatment as a risk factor for studied autoimmune diseases in full term children, it has been shown to modulate neonate’s gut microbiota [48]. Conclusively, postnatal antibiotic treatments and other preterm-related factors (gut immaturity, intrauterine infection, maternal antibiotics, type of early feeding, etc.) could influence the composition of gut microbiota in preterm neonates and predispose dysbiosis [49]. It is uncertain, whether (such) dysbiosis during neonatal period might extend beyond early childhood and associate with the onset of DM, AIT, JIA, and/or IBD [50–55]. Therefore, long-term studies on gut microbiome involving pre-diagnostic phase of these autoimmune diseases are warranted.

In our cohort, high maternal employment and low percentage of maternal smoking (7.5%) corresponded to previous reports [56, 57], and most importantly, were similar for all children. These aspects preclude the estimation of how heavy maternal smoking and/or lower socioeconomic status, often intertwining with one another, would relate to the development of autoimmune diseases. Previous studies relating intrauterine exposure to smoking and autoimmune diseases have presented various results as well [58–61]. However, these studies have not always considered the role of preterm birth, which was more common among smoking women [62].

The strength of our study lies in the coverage of the cohort and the comprehensive data on the national registers. With high worldwide incidence of pediatric autoimmune diseases, Finland is an eligible representative for studies on their environmental risk factors. Our study population was vastly distributed throughout the country, enclosing all densely populated areas. Furthermore, the registration coverage of the Finnish public health system is exceptional. All chronic autoimmune diseases are diagnosed and treated in the public sectors, and utilized data are submitted to nationwide registers with confirmed validity [17]. By using these registers, our data was assured to be comprehensive. Nevertheless, since our data was mostly obtained from densely populated regions, comparison between urban and rural areas was unfeasible—this was one of our limitations. Moreover, ethnic variations in Finland are very small, and most residents are highly educated. This is especially true concerning parents in our cohort, which implies a possible bias toward participants from families with high education level [16]. Lack of diversity in social background of our study population may limit observations on certain risk factors related to it such as socioeconomic status, smoking habits, and role of ethnical backgrounds. Other limitation concerns variations in the children’s age at the end of follow-up. This might especially influence AIT, which peak incidence occurred at later age than other studied autoimmune diseases. We estimated that the number of new AIT cases would have increased slightly if all children had been followed until the age of 18, resulting to AIT incidence of 50.2, but this did not alter our conclusion about it. Finally, we focused only on four pediatric autoimmune diseases. Celiac disease has a known triggering factor (gluten) and requires no long-term medical prescriptions, thus was not available in SRR [63]. Asthma was excluded due to different criteria for receiving special reimbursement (a previous non-stop medical treatment of minimum 6 months is required), and many infants outgrow their obstructive symptoms and the need for medications.

## Conclusion

We have discovered that preterm birth and postnatal antibiotic use associated with it are related to typical pediatric onset autoimmune diseases (DM, AIT, JIA, and IBD. These perinatal factors were not related to any particular disease, hence could be a shared risk factor. Furthermore, high maternal age was related to IBD. Our findings may provide partial explanation for the high incidence of these pediatric autoimmune diseases, but further studies are required to focus on possible mechanisms in details, such as the stability of neonatal gut dysbiosis.

## Supplementary Information

ESM 1 (DOCX 21 kb)

## Data Availability

Data available upon request.

## Abbreviations

AIT: Autoimmune thyroiditis
ATC: Anatomical therapeutic chemical
CD: Crohn disease
DM: Type 1 diabetes
DPR: Drug purchase register
IBD: Inflammatory bowel diseases
IBDU: Inflammatory bowel diseases unclassified
IQR: Interquartile range
JIA: Juvenile idiopathic arthritis
MBR: Medical Birth Register
SD: Standard deviation
SRR: Special Reimbursement Register
UC: Ulcerative colitis
