# Supplementary material for: Perinatal risk factors for pediatric onset type 1 diabetes, autoimmune thyroiditis, juvenile idiopathic arthritis, and inflammatory bowel diseases
Source: Eur J Pediatr. 2021 Feb 23;180(7):2115–23. doi: 10.1007/s00431-021-03987-3 (PMC8195774; doi:10.1007/s00431-021-03987-3)
Supplement: Supplementary file 1 — (DOCX 21 kb) [file 431_2021_3987_MOESM1_ESM.docx]

**Supplemental tables**

**Supplement table 1:** Incidence of pediatric onset DM, AIT, JIA, and IBD^a^ per 100,000 children per year with upper 95% CI. Additional data for figure 1.

|  | **Incidence/100,000 children/year (95% CI^b^) in different age groups** | | |  |  | |
| --- | --- | --- | --- | --- | --- | --- |
| **Diagnosis** | **Preschool age**  **(0-5.9 years)** | **Pre-pubertal age**  **(6-11.9 years)** | **Adolescence**  **(12-18.9 years)** | | |  |
| **DM** | 52.6 (38.9-72.8) | 59.9 (44.2-81.2) | 36.5 (24.8-54.2) | | |  |
| **AIT** | 4.4 (1.5-12.9) | 30.7 (20.1-46.9) | 64.3 (47.9-86.4) | | |  |
| **JIA** | 29.2 (18.9-45.1) | 26.3 (16.6-41.6) | 24.8 (15.5-39.8) | | |  |
| **IBD** | 5.8 (2.3-15.0) | 16.1 (9.0-28.8) | 20.5 (12.2-34.3) | | |  |

^a^ Abbreviations: DM= type 1 Diabetes Mellitus, AIT= Autoimmune Thyroiditis, JIA= Juvenile Idiopathic Arthritis, IBD= Inflammatory Bowel Diseases

^b^ CI= Confidence Interval

**Supplement table 2.** Sex distribution of DM, AIT, JIA, and IBD^a^ per 100,000 children per year. The children were born 2000-2005. Additional data for figure 2.

|  | **Incidence/100,000 children/year (95% CI^b^)** | |  | |  | |
| --- | --- | --- | --- | --- | --- | --- |
| **Diagnosis** | **Boys** | **Girls** | | **Total** | |  |
| **DM** | 65.5 (51.2-83.7) | 40.6 (29.7-55.4) | | 106.1 (87.5-128.6) | |  |
| **AIT** | 13.5 (5.8-20.9) | 32.4 (24.5-43.0) | | 46.0 (36.3-58.2) | |  |
| **JIA** | 11.0 (6.2-19.7) | 44.0 (32.8-59.1) | | 55.0 (42.3-71.6) | |  |
| **IBD** | 13.1 (8.0-21.2) | 10.6 (6.2-18.2) | | 23.7 (16.5-34.0) | |  |

^a^ Abbreviations: DM= type 1 Diabetes Mellitus, AIT= Autoimmune Thyroiditis, JIA= Juvenile Idiopathic Arthritis, IBD= Inflammatory Bowel Diseases

^b^ CI= Confidence Interval

**Supplement table 3a.** Frequency of DM, AIT, JIA, and IBD^a^ in relation with maternal backgrounds, delivery method, and perinatal factors in preterm (born <37 weeks) children.

|  | **Preterm children with primary diagnoses**  **(N=21)** | **Preterm children without diagnoses**  **(N=593)** | **p-value** |
| --- | --- | --- | --- |
| **Maternal employment status (%)** |  |  | 0.354^b^ |
| - Upper-level employees | 8 (38.1) | 160 (27.0) |  |
| - Lower-level employees | 5 (23.8) | 270 (45.5) |  |
| - Manual and self-employed workers | 4 (19.0) | 68 (11.4) |  |
| - Students | 3 (14.3) | 59 (9.9) |  |
| - Housewifes | 1 (4.8) | 23 (3.9) |  |
| - Unemployed, pensioners | 0 | 1 (0.2) |  |
| - Missing data | 0 | 12 (2.0) |  |
|  |  |  |  |
| **Mother’s smoking habit (%)** |  |  | 0.922^b^ |
| - Not smoking | 19 (90.5) | 497 (83.8) |  |
| - Quitted before 1^st^ trimester | 0 | 10 (1.7) |  |
| - Continued after 1^st^ trimester | 1 (4.8) | 57 (9.6) |  |
| - Missing data | 1 (4.8) | 29 (4.9) |  |
|  |  |  |  |
| **Maternal age, mean ±SD** | 32.4±6.1 | 30.9±5.4 | 0.227^d^ |
|  |  |  |  |
| **Parity, median**  **(IQR)**  **(Range)** | 0  (0-2)  (0-2) | 0  (0-1)  (0-8) | 0.693^e^ |
|  |  |  |  |
| **Delivery method (%)** |  |  | 0.581^b^ |
| - Normal vaginal delivery | 10 (47.6) | 321 (54.1) |  |
| - Forceps or vacuum extraction | 1 (4.8) | 19 (3.2) |  |
| - Cesarean section | 10 (47.6) | 252 (42.5) |  |
| - Missing data | 0 | 1 (0.2) |  |
|  |  |  |  |
| **Birthweight (g), mean ± SD** | 2226±667 | 2403±673 | 0.237^d^ |
| **Birth height (cm), mean ± SD** | 44.6±4.7 | 45.3±3.8 | 0.460^d^ |
| **Postnatal antibiotic treatments (%)** | 10 (47.6) | 164 (27.7) | 0.046^c^ |
| **Inpatient length of stay (days), median**  **(range)** | 5  (2-39) | 5  (0-71) | 0.824^e^ |

^a^ DM=Type 1 Diabetes Mellitus, AIT=Autoimmune Thyroiditis, JIA=Juvenile Idiopathic Arthritis, IBD=Inflammatory Bowel Diseases

^b^ Fisher’s exact test; ^c^ Chi-square test; ^d^ Independent Samples t-test; ^e^ Kruskall-Wallis H test

**Supplement table 3b.** Postnatal antibiotic treatments in children who developed DM, AIT, JIA, and IBD^a^

| **Diagnosis/**  **Preterm children (< 37 weeks)** | **DM**  **(N=9)** | **AIT**  **(N=4)** | **JIA**  **(N=4)** | **IBD**  **(N=4)** | **p-value^b^** |
| --- | --- | --- | --- | --- | --- |
| **Postnatal antibiotic treatments (%)** |  |  |  |  | 0.164 |
| - Yes | 4 (44.4) | 1 (25.0) | 3 (75.0) | 2 (50.0) |  |
| - No | 5 (55.6) | 3 (75.0) | 1 (25.0) | 2 (50.0) |  |
|  |  |  |  |  |  |
| **Diagnosis/**  **Full term children (≥ 37 weeks)** | **DM**  **(N=82)** | **AIT**  **(N=62)** | **JIA**  **(N=50)** | **IBD**  **(N=25)** | **p-value^b^** |
| **Postnatal antibiotic treatments (%)** |  |  |  |  | 0.750 |
| - Yes | 1 (1.2) | 2 (3.2) | 2 (4.0) | 1 (4.0) |  |
| - No | 81 (98.8) | 60 (96.8) | 48 (96.0) | 24 (96.0) |  |
|  |  |  |  |  |  |
| **Missing cases (not included)** | 11 | 2 | 4 | 0 |  |

^a^ Abbreviations: DM= type 1 Diabetes Mellitus, AIT= Autoimmune Thyroiditis, JIA= Juvenile Idiopathic Arthritis, IBD= Inflammatory Bowel Diseases

^b^ Fisher’s exact test
